# Post-traumatic Growth and Psychological Resilience During the COVID-19 Pandemic: A Serial Mediation Model

**DOI:** 10.3389/fpsyt.2022.780807

**Published:** 2022-03-21

**Authors:** Qi Li, Jinsheng Hu

**Affiliations:** Department of Psychology, Liaoning Normal University, Dalian, China

**Keywords:** post-traumatic growth, psychological resilience, positive coping, cognitive reappraisal, college students

## Abstract

Although the World Health Organization (WHO) has issued guidelines for managing to contain, mitigate, and limit of the COVID-19. However, it is more essential to highlight the urgency and importance of evaluating social functioning and mental health status during the pandemic. College students have experienced serious problems and have had to overcome many negative situations brought about by the pandemic. Accordingly, the present study intended to use Chinese college students as sample to examine the positive adoption and changes during the ongoing COVID-19. Guided by literatures in this filed, we explored the internal mechanism of post-traumatic growth affecting psychological resilience, and considered about mediation roles of positive coping styles and cognitive reappraisal. A total of 463 college students from universities in China effectively completed online questionnaires. The result indicated that these four variables were positively correlated with each other (*p*s < 0.001). More importantly, our findings proved a direct and positive effect on psychological resilience. Positive coping styles and cognitive reappraisal, respectively, mediated the relationship between post-traumatic growth and psychological resilience. Over all, the hypothesized serial model conclusively fits the data: students with high-level post-traumatic growth tended to report increased use of positive coping strategies, which further facilitated their cognitive reappraisal, and subsequently, promoted their psychological resilience. The findings obtained in this study will provide a theoretical basis and possible viable strategies for both targeted crisis intervention and psychological trauma recovery plans.

## Introduction

The novel coronavirus disease (COVID-19) is a respiratory disease characterized by high transmissibility and mortality rates (1). On January 30, 2020, the World Health Organization (WHO) declared the outbreak of a public health emergency of international concern. As this pandemic continues to spread with strong momentum and a lack of specific treatment, restrictions on social contact (large-scale lockdowns), and appropriate infection prevention strategies (travel limitation, quarantine, and self-isolation) have been implemented to control virus propagation. Obviously, both the COVID-19 emergency and the extraordinary measures to contain it have had a profound and wide range of psychosocial impact (2). More and more experts have highlighted the urgency and importance of evaluating social functioning and mental health status during the COVID-19 pandemic.

In response to the outbreak of infection, the Chinese government and health agencies have ordered nationwide school closures as an emergency measure to control the pandemic. Students in higher education experience multifaceted pressures, such as academic workload, economic difficulties, relationship issues, and employment concerns brought about by the pandemic (3). These stressful experiences may put college or university students at a high risk of future mental health problems (4, 5). However, negative experience can be a “catalyst” for positive change. Recovery from initial heightened levels of distress may be more common, particularly when adversity involves extensive disruptions over an extended period of time (6). Given the ongoing nature of the COVID-19 pandemic, people in their respective regions need to quickly adjust their minds and lifestyles and adapt to the “new normal.” Hence, it is imperative to identify factors that may have protective effects on the physical and mental status of college students.

The American Psychological Association defines psychological resilience (PR) as “a process of good adaptation in the face of adversity, trauma, tragedy, threats, or other significant sources of stressors such as family and relationship problems, serious health problems, or financial problems” (7). In general, PR is a context-dependent “reconfiguration,” refers to an individual’s ability to quickly rally, recover, and return to their pre-crisis status after trauma exposure (8). Similarly, PR is also viewed as a measure of stress coping ability in response to adversity, which can help individuals reduce their vulnerability to challenges and difficulties (9). Focus on university and college students, studies have documented that improving PR can cushion the psychological trauma caused by stress and can contribute to students’ academic success (10), improve their sense of well-being (11), and enable them to effectively cope with stressful situations (12).

With regard to COVID-19, PR was identified as a negative predictor of depression, anxiety, and somatization in the general population during the peak of the pandemic in China (13). The available data suggests that PR plays a protective role against the fear of COVID-19, individuals with high psychological resilience experience fear less (14). In fact, these studies provide evidence on psychological resilience factors, such as protective factors for mental health disorders, have been extensively discussed (15). However, the specific mechanism of PR as the outcome variable has scarcely been investigated, especially among college students in the context of the COVID-19 pandemic.

### Post-traumatic Growth and Psychological Resilience

Although traumatic events alter daily life to a certain extent, people who have been confronted with trauma not only have post-traumatic negative symptoms but may also undergo positive psychological changes. Post-traumatic growth (PTG) is one of the most widely discussed salutogenic post-traumatic consequences, which is defined as “positive psychological change experienced as a result of the struggle with highly challenging life circumstances” (16). The occurrence of PTG can help individuals think more and take coping measures more proactively after experiencing traumatic events. Indeed, PTG enables individuals to reframe their experiences and perceive meaningful personal growth potential from a major life crisis, which can improve relationships with others, create new possibilities, enhance personal strength, bring spiritual development, or increase appreciation of life (17). These deep reconsiderations are what will facilitate individuals to establish new life goals. The maintenance of the growth experienced may require unpleasant periodic cognitive reminders of what has been lost, so that in an apparently paradoxical way, what has been gained remains in focus (18). Therefore, this process can sometimes take months or years and, in some cases, as PTG also depends on personal traits, individuals who have been confronted with trauma may never experience positive psychological changes (19). As both psychological states emphasize transformation after a trauma, PTG is considered to have a close correlation with PR (19, 20). In fact, growth is not a result of the event itself, but rather a result of the struggle to deal with it, which means that PTG not only requires one’s post-traumatic response and relief, but also helps to enhance oneself and become stronger (21).

As aforementioned, individuals with higher levels of PTG feel powerful enough to handle problems in their life and can easily adapt to traumatic events by focusing on positive outcomes, as they possess improved coping mechanisms and improved psychological well-being (22). In this regard, the abilities and resources possessed before the event should receive more attention. PTG ensures a deeper perspective and strength to people after traumatic events, which contributes to stronger beliefs and ability for action, increasing psychological resistance to negative effects when dealing with subsequent adversity (16, 23). Theoretically, experience and positive changes after trauma can be antecedent factors for the development of PR. However, no empirical research has confirmed the mechanisms of PTG with regard to PR systematically. The current study aimed to test whether PTG of college students can predict PR positively in the context of the COVID-19 epidemic. Thus, the following hypothesis is proposed:

Hypothesis 1: PTG is directly associated with PR.

Post-traumatic growth emphasizes individuals’ transformation in the aftermath of stressful events that may reshape their assumptive world, requiring a reconceptualization of fundamental beliefs about the self, others, and the future (24). Several studies have suggested that positive sense-making, positive reframing/reinterpretation, and positive affect/attitude are positively correlated to PTG (19, 25). We suggest that growth from traumatic events may provide individuals with more positive coping strategies and effective emotion regulation to deal with subsequent trauma, which could play an essential role in fostering PR. Even so, there remains a dearth of in-depth studies on the serial mediating mechanism of “PTG-PR.”

### The Mediating Role of Coping Style

Coping style has been identified as a process of managing external or internal demands and an important intermediary regulating factor in the process of psychological stress (26). Some coping styles involve adaptive or constructive coping strategies, such as seeking support and trying to change, while some are mainly considered maladaptive, such as avoidance and venting (27). In accordance with this viewpoint, Xie proposed to divide coping styles into positive coping (PC) styles and negative coping (NC) styles (28). The former helps to buffer the impact on individuals and maintain both physical and mental health (29), whereas the latter plays a contrary role (30).

Coping involves using behavioral, cognitive, and emotional strategies to handle and manage stressful events or negative psychological outcomes, whereas resilience refers to the adaptive capacity to recover from adversity and the successful final result of effectively implementing these strategies (31). However, the nature of the relationship between resilience and coping style has not yet been clearly established. One study found that coping style mediates the relationship between resilience and psychological well-being (32), while other studies have observed that coping style predicts resilience (33, 34). Using of coping strategies such as positive reinterpretation appears to be a resilience-building intervention. Such interventions provide opportunities to exercise and develop adaptive coping responses with appropriate scaffolding and guidance, which is integral to realize one’s resilience potential.

A higher level of positive coping styles may be related to increased levels of positive cognitive and behavioral adjustments in the face of trauma. A burgeoning body of literature have proved that utilization of PC mechanisms such as seeking social support, positive thinking, and problem solving was associated with lower levels of traumatic stress, stigma (35). Moreover, one study demonstrated that PTG was predictive of hope and PC but negatively predictive of anxiety (36). Although there has been limited investigation of direct effects of PC on the PR of college students during this major public health event, based on these previous findings, it can be inferred that PTG is indirectly associated with PR via PC. Thus, the following hypothesis is proposed:

Hypothesis 2: PC mediates the relationship between PTG and PR.

### The Mediating Role of Emotion Regulation

Emotion regulation (ER) is identified as the cognitive behavioral process whereby individuals consciously and/or non-consciously adjust internal affective states to respond to environmental demands appropriately, thereby generating adaptive responses (37, 38). An accumulating body of evidence indicates that enhanced ER is positive related to PR and that discrete ER strategies (e.g., reappraisal and suppression) are likewise related to PR (39, 40). In particular, the positive association between cognitive reappraisal (CR) and PR was confirmed in six studies, even when controlling for other explanatory variables (41–46). In the same way, it can be easily proposed that PTG is indirectly associated with PR via CR. Thus, the following hypothesis is proposed:

Hypothesis 3: CR mediates the relationship between PTG and PR.

Notably, improved coping capacity further enhances PR by contributing to emotion-and problem-focused coping during and after negative experiences (47). The relationship between PC and CR is not yet clear; however, their common core emphasizes that protective factors provide to individuals facing adversity determines the existence and development of their PR. In this case, the enhancement of PR after trauma lies not in avoiding stress but in how people adopt positive solving means and emotion regulation strategies to handle it. Therefore, we offer the following hypothesis:

Hypothesis 4: PTG can affect PR through the serial mediating roles of PC and CR.

### The Current Study

**FIGURE 1:**
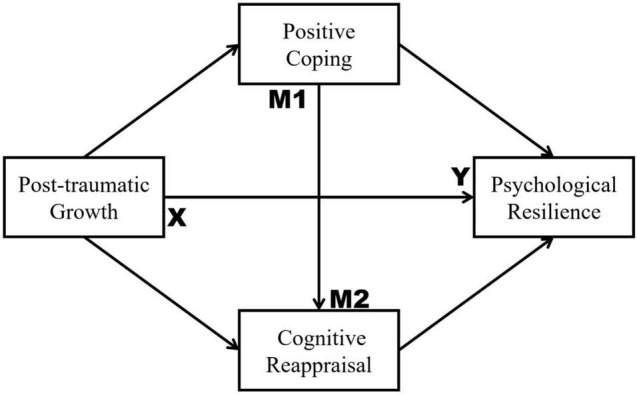
Proposed research model.

The WHO has issued guidelines for managing containment, mitigation, and limiting of COVID-19. However, it is our contention that psychological preventive and therapeutic measures are just as crucial in facing the pandemic. College students have experienced serious problems and have had to overcome many negative situations during this period (48). Accordingly, the present study used Chinese college students as sample to observe positive characteristics adoption and changes during the ongoing COVID-19 pandemic. Boosting mental resilience can help to successfully deal with the coronavirus pandemic. The findings obtained in this study will provide a theoretical basis and possible viable strategies for both targeted crisis intervention and psychological trauma recovery plans. For instance, psychologists or other professionals could be called on to provide psychological education or rapid and easy-to-access interventions (e.g., online counseling or hotlines), aimed at guiding effective coping behavior and positive emotional cognition in the face of this and other potential public health emergencies. To this end, we aimed to propose a serial mediation model and test the internal mechanism of PTG affecting PR, and in particular, to examine the mediating roles of PC and CR in Chinese college students during the COVID-19 pandemic (see Figure 1).

## Materials and Methods

### Sampling Procedure and Sociodemographic Background

The present study is a cross-sectional analysis that used convenience sampling methods. Convenience sampling is a non-probability sampling technique where subjects are selected because of their convenient accessibility and proximity to the researcher. The online survey was conducted between May 18 and July 22, 2021, by distributing QR codes to full-time undergraduate students in mainland China through the Wenjuanxing platform^1^. Before participating in the survey, all subjects were informed of the nature of the study and provided informed consent. They were also assured that personal information would not be shared with others without their permission. The recruitment and data collection procedures were approved by the Ethics Committee of Liaoning Normal University.

Participants were asked to complete a battery of online questionnaires. Only after completing all items was the questionnaire submitted. Otherwise, the assessment system automatically recorded the data as incomplete. The test time was set by pretest results, and questionnaires with test times of less than 4 min were deleted. Excluding invalid questionnaires from 24 participants, the final sample size was 463. Among 463 respondents, 365 (78.83%) were female. The majority (78.2%) were 2nd and 3rd year undergraduates, 18.8% were first year undergraduates. Approximately one in five of the sample were medical students. Over a half were from only child families. Only 40.6% lived in rural areas.

To ensure the statistical power and effect size, we conducted a *post hoc* statistical power calculator (G*Power, version 3.1.9.7; Heinrich Heine University Düsseldorf) with a medium effect size (*r* = 0.3), the desired statistical power level of 0.8, and significance level (α) of 0.05. The result showed that the observed power (1-β) for two-tailed hypothesis was 0.99, indicating an acceptable power and effect size.

### Materials

Instructions were provided at the beginning of the questionnaire, while general data included sociodemographic factors, the COVID-19 awareness and the impact of the pandemic. Sociodemographic demographic details covered gender, grade, residence, major (medical, non-medical), and only-child status or not. Awareness and impact of COVID-19 were collected through: “How much you care about your physical/mental health during this outbreak,” “Your recognition of the effectiveness of the epidemic prevention and control measures in China,” and “Your assessment of the future epidemic situation in China.” The previous three questions were assessed using a 5-point Likert scale ranging from 1 (none) to 5 (very significant). Besides, data about the online psychological assessments were composed of four scales: (a) post-traumatic growth inventory, (b) psychological resilience scale, (c) simplified coping style questionnaire, and (d) emotion regulation questionnaire.

#### Post-traumatic Growth Inventory

The Post-traumatic Growth Inventory was developed by Tedeschi and Calhoun to assess the positive experiences of individuals who have experienced traumatic events (49). The Chinese version adapted to suit the Chinese context has demonstrated adequate psychometric properties. The 21-item scale is divided into five dimensions, including relationship to others (seven items), new possibilities (five items), personal strength (four items), spiritual change (two items), and appreciation of life (three items). Each item is scored on a 6-point Likert scale ranging from 0 (no change) to 5 (complete change). The total PTG score was the sum of all item scores. A higher score represents additional positive psychological changes in the aftermath of the trauma. The PTG scale here was emphasized to response considering the COVID-19 epidemic context by specific description. In the current study, Cronbach’s Alpha was 0.96 and McDonald’s Omega was 0.96.

#### Psychological Resilience Scale

The Connor-Davidson Resilience Scale (CD-RISC) is considered a reliable and valid instrument for evaluating psychological resilience (50). The adapted Chinese version of the CD-RISC measures personal resilience over the past 30 days and has adequate content validity, internal consistency, and test-retest reliability (51). This revised version is divided into three factors: optimism (four items), strength (eight items), and tenacity (13 items). Participants respond to 25 items using 5-point scoring, ranging from 0 (never) to 4 (always). Consequently, the total score can be obtained by adding up the responses (values) of all items, ranging from 0 to 100. Higher scores reflect a greater degree of psychological resilience. In the current study, Cronbach’s Alpha was 0.91 and McDonald’s Omega was 0.92.

#### Simplified Coping Style Questionnaire

The Simplified Coping Style Questionnaire (SCSQ, Chinese version) was developed by Xie to assess participants’ attitudes and coping styles regarding specific life events or difficulties encountered in their daily lives (28). A total of 20 items encompasses two dimensions: items 1–12 describe positive coping styles (e.g., “to be free from work, study, or some other activities”) and items 13–20 describe negative coping styles (e.g., “relieve trouble by smoking, drinking, taking medicine, and holding things”). Each item is rated on a 4-point Likert scale ranging from 0 (never) to 3 points (very often). The higher the dimension score, the more habitually the corresponding coping style is used by individuals with stress. Based on the hypotheses, only positive coping styles were utilized in the mediation model. In the current study, Cronbach’s Alpha was 0.85 and McDonald’s Omega was 0.85.

#### Emotion Regulation Questionnaire

The Emotion Regulation Questionnaire was developed by Gross and John to assess ER strategies (52). The Chinese translations of these items were adopted (53). The questionnaire consists of 10 items rated on a 7-point scale ranging from 1 (strongly disagree) to 7 (strongly agree), which have two opposing dimensions. Six items measured the degree of cognitive reappraisal (e.g., “When I wanted to feel less negative emotion, I changed the way I was thinking about the situation”), and four items measured the degree of expressive suppression (e.g., “I controlled my emotions by not expressing them”). The total score ranges from 10 to 70, with higher scores reflecting greater use of the specific emotion regulation strategy. Based on the hypotheses, only cognitive reappraisal was utilized in the mediation model. In the current study, Cronbach’s Alpha was 0.82 and McDonald’s Omega was 0.82.

### Statistical Analysis

Upon completion of data collection, all analyses were performed utilizing SPSS 28.0 (IBM Corp., Armonk, NY, United States). First, the original questionnaire data needed to be preliminary analysis. Common method variance (CMV) is one of the main sources of measurement error threatens the validity of the conclusions about the relationships between measures. CMV means that variance is attributable to the measurement method rather than to the constructs the measures represent (54). Thus, we undertook procedural and process remedies to control the effect of CMV (55). On the one hand, an *ex ante* approach was adopted in the design of questionnaires (56). Participants were assured confidentiality and anonymity by an introductive message to minimize social desirability effect (57). On the other hand, Harman’s single-factor test (SPSS 28.0 version) and the method-factor approach (Mplus 8.3 version) were conducted to ensure statistical control. Harman’s test assumes that if CMV is presented in the data, one variable will account for more than 40% of the covariance in the independent and dependent variables (54, 58). In other words, the variation between the independent and dependent variables was caused more by the methods of data collection and measurement than by difference in the nature of variables. Second, the internal consistency for each measure was checked by reliability tests using Cronbach’s Alpha and McDonald’s Omega coefficient (59). Third, for the sociodemographic variables and awareness of COVID-19, descriptive statistics were reported as means and standard deviations for continuous variables and percentages for categorical variables. Fourth, Spearman’s correlation analysis was conducted to examine the relationships between four variables. Finally, as shown in Figure 1, the serial mediation model posits how, or by what means, one predictor (X) affects one outcome variable (Y) through two potential mediators (M1 and M2). Three models were fitted: regressing the first mediator on the predictor, regressing the second mediator on the first mediator and the predictor, and regressing the outcome on all mediators and the predictors (60). Moreover, our study used bootstrapping, a non-parametric resampling procedure, to test hypotheses about mediation. Bootstrapping involves resampling the raw data and forming an empirical distribution of the indirect effect point estimates to form the confidence interval (CI) of an indirect effect (60, 61). Compared with conventional methods, this method has higher power while maintaining reasonable control over the type I error rate (62). Repeated sampling 5,000 times were generated from the original sample set (*N* = 463) to calculate the 95% CI. The absence of zero in the 95% CI of the standardized path coefficient indicated that the mediation effects were significant. The hypotheses were tested using the SPSS plug-in PROCESS macro program (version 3.4). All differences were considered statistically significant at *p* < 0.05 (two-sided).

## Results

### Common-Method Variance Test

An explanatory factor analysis (EFA) including all variables using unrotated principal components factor analysis was performed to statistically verify the presence of CMV (54). The results revealed that 12 factors had eigenvalues greater than 1, and the general factor accounted for only 27.29% of the total variance, which did not exceed the critical value of 40%. Furthermore, a confirmatory factor analysis (CFA) was also carried out. All items are allowed to load on their theoretical constructs, as well as on an unmeasured latent CMV factor (adding a first-order factor with all of the measures as indicators to the theoretical model), and the significance of the structural parameters is examined both with and without the latent CMV factor in the model. The results showed that the goodness-of-fit was not significantly improved after adding the common method factor (χ^2^/df = 2.800, *CFI* = 0.770, *RMSEA* = 0.062, *TLI* = 0.761, *SRMR* = 0.228) to the four-factor model of this study (χ^2^/df = 2.674, *CFI* = 0.786, *RMSEA* = 0.060, *TLI* = 0.778, *SRMR* = 0.059). All these tests concluded that CMV was not a concern.

### Socio-Demographic Variables and Awareness of COVID-19

The characteristics regarding the sociodemographic data and self-assessment about COVID-19 of the study population are shown in Table 1. Regarding the awareness of the pandemic, participants concerned more about their physical and mental health during the epidemic and showed a positive attitude toward the situation. Most of them are satisfied with local pandemic prevention measures and not worried about the future epidemic situation in China.

**TABLE 1:** Socio-demographic characteristics and awareness of the COVID-19.

Category	Subcategory	*N* (%)	Concern (Mean ± SD)	Satisfaction (Mean ± SD)	Estimation (Mean ± SD)
Gender	Female	365 (78.8%)	3.17 ± 1.01	4.40 ± 0.85	1.72 ± 0.65
	Male	98 (21.2%)	3.01 ± 1.17	4.47 ± 0.86	1.64 ± 0.68
Grade	1	87 (18.8%)	3.07 ± 1.08	4.46 ± 0.79	1.69 ± 0.56
	2	211 (45.6%)	3.31 ± 1.03	4.42 ± 0.85	1.81 ± 0.67
	3	151 (32.6%)	2.97 ± 1.01	4.38 ± 0.89	1.59 ± 0.66
	4	10 (2.2%)	2.60 ± 0.70	4.30 ± 1.06	1.30 ± 0.48
	5	2 (0.4%)	3.00 ± 2.83	4.50 ± 0.71	2.00 ± 1.41
	Other	2 (0.4%)	3.00 ± 2.83	4.50 ± 0.71	1.50 ± 0.71
Major	Medical	95 (20.5%)	2.84 ± 1.03	4.39 ± 0.89	1.55 ± 0.61
	Non-medical	368 (79.5%)	3.21 ± 1.04	4.42 ± 0.84	1.75 ± 0.66
Residence	Urban	275 (59.4%)	3.11 ± 1.06	4.42 ± 0.80	1.73 ± 0.67
	Rural	188 (40.6%)	3.18 ± 1.04	4.40 ± 0.93	1.67 ± 0.63
Family	Only child	257 (55.5%)	3.17 ± 1.06	4.40 ± 0.84	1.75 ± 0.66
	Multiple-children	206 (44.5%)	3.09 ± 1.04	4.43 ± 0.87	1.65 ± 0.64

*(N = 463).*

### Descriptive Statistics and Correlation Analysis

Descriptive statistics and a correlation matrix of the study variables are given in Table 2. The Shapiro–Wilk normality test found that only PR data is normally distributed (*p* > 0.05). Hence, the present data was described by first quartile (Q1), Median and third quartile (Q3), and was calculated using Spearman’s correlation analysis. Bivariate correlation analyses showed that PR, PC, CR, and PTG were positively correlated with each other (*p*s < 0.001). Results of the Spearman correlations between research variables are generally in line with our expectation, which met the prerequisites for subsequent research hypotheses and conducting mediation testing.

**TABLE 2:** Descriptive statistics and correlation coefficients.

	*Median (Q1*, *Q3)*	PR	PC	CR
PR	2.44 (2.16, 2.80)			
PC	2.00 (1.75, 2.33)	0.58***		
CR	5.50 (4.83, 6.00)	0.41***	0.42***	
PTG	2.62 (1.95, 3.23)	0.51***	0.49***	0.29***

*(N = 463).*

*PR, psychological resilience; PC, positive coping; CR, cognitive reappraisal; PTG, post-traumatic growth.*

*p < 0.05, **p < 0.01, ***p < 0.001.

### Serial Mediation Model

**FIGURE 2:**
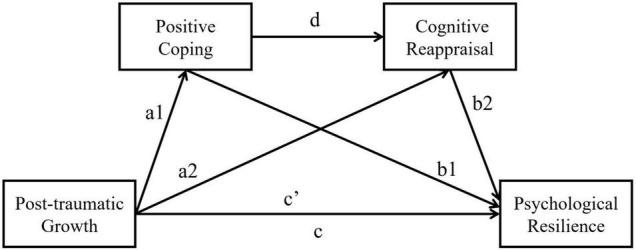
Roadmap of the influence of post-traumatic growth on psychological resilience.

For further testing the mediation effects, a serial mediation model was conducted with PROCESS (model 6) for SPSS, using with PTG as predictor, two serial mediators (PC→CR), and PR as outcomes. The conceptual model (see Figure 2) is based on three linear regression analyses. The first regression analysis tests the effects of PTG on PC (path a1). The second regression model tests the combined predictive effects of PTG and PC on CR (paths a2 and d). The third regression predicts the PR by the independent variable PTG and the two mediators (paths b1, b2, and c’). Here, path c’ depicts the direct effect of PTG on the PR controlled for the effects of the two mediators. In contrast, path c indicates the total effect of PTG on PR without considering the mediators.

Model indices are depicted in Table 3. In the path of a1→b1, PTG had a significant positive effect on PC (β = 0.22, *p* < 0.001), while PC had a significant positive effect on PR (β = 0.42, *p* < 0.001). Thus, PTG enhanced PR by adopting more positive coping strategies. In the path of a2→b2, PTG had a significant positive effect on CR (β = 0.09, *p* < 0.05), while CR had a significant positive effect on PR (β = 0.11, *p* < 0.001). Thus, PTG improved PR also by increased reappraisal emotion regulation. In the path of a1→d→b2, PC had a significant positive effect on CR (β = 0.64, *p* < 0.001). This indicates that the PC was closely related to students’ CR. Furthermore, PTG enhanced the CR by increase PC, which finally increased students’ PR. These results supported hypotheses 1–4.

**TABLE 3:** Regression results for mediation analysis.

Model	Outcome	Predictors	β	*SE*	*t*	LLCI	ULCI
Model 1	PC	Constant	1.43	0.06	26.00***	1.33	1.54
		PTG	0.22	0.02	11.00***	0.18	0.26
	*R^2^* = 0.21, *F* = 121.06***						
Model 2	CR	Constant	3.91	0.15	26.06***	3.61	4.20
		PTG	0.09	0.04	2.16*	0.01	0.16
		PC	0.64	0.08	7.92***	0.48	0.80
	*R^2^* = 0.19, *F* = 52.42***						
Model 3	PR	Constant	0.78	0.11	6.76***	0.55	1.00
		PTG	0.10	0.02	5.37***	0.07	0.14
		PC	0.42	0.04	10.15***	0.34	0.51
		CR	0.11	0.02	4.64***	0.06	0.15
	*R^2^* = 0.43, *F* = 116.01***						

*PR, psychological resilience; PC, positive coping; CR, cognitive reappraisal; PTG, post-traumatic growth; LLCI, boot CI lower limit; ULCI, boot CI upper limit.*

*p < 0.05, **p < 0.01, ***p < 0.001.

For the prediction of PR (Table 4), PTG was a statistically significant and positive predictor (*c* = 0.22, *p* < 0.001) in the total effect model without consideration of the mediators. However, the explained variance increased by Δ*R*^2^ = 0.21 when the mediators, PC and CR, were included in the model. All three possible indirect effects were significant [a1→b1: *b* = 0.10, 95% CI (0.07–0.13); a2→b2: *b* = 0.01, 95% CI (0.00–0.02); a1→d→b2: *b* = 0.02, 95% CI (0.01–0.02)]. Correspondingly, the total indirect effect was significant [*b* = 0.12, *p* < 0.001; 95% CI (0.09–0.15)], whereas the direct effect was reduced by inclusion of the mediators but remained significant, too [*c*’ = 0.10, *p* < 0.001; 95% CI (0.07–0.14)]. The effect size of the mediating pathways was calculated using the formula (ab)/c, showed that path a1→b1, a2→b2 and a1→d→b2 accounted for 42.62, 4.02, and 6.75% of the total effect, respectively.

**TABLE 4:** Effects and 95% confidence intervals for Model 3.

	Effect	*SE*	*t*	LLCI	ULCI
Total effect	0.22	0.02	11.06***	0.18	0.26
Direct effect	0.10	0.02	5.37***	0.07	0.14
PTG→PC→PR	0.10	0.01	–	0.07	0.13
PTG→CR→PR	0.01	0.00	–	0.00	0.02
PTG→PC→CR→PR	0.02	0.00	–	0.01	0.02

*PR, psychological resilience; PC, positive coping; CR, cognitive reappraisal; PTG, post-traumatic growth; LLCI, boot CI lower limit; ULCI, boot CI upper limit.*

*p < 0.05, **p < 0.01, ***p < 0.001.

## Discussion

People can be at the greater risk of developing various mental health problems following traumatic stress (63). The identification of protective factors of mental health problems is vital to improve the well-being and the psychological health of individuals when facing difficulties (15, 64). What remains to be answered is whether PTG plays a positive role in PR and how PTG contributes to PR during the later pandemic period among Chinese college students. According to the thorough review on previous studies, both PC and CR are effective protective factors related to enhanced PR (33, 34). However, the aforementioned four concepts have not been handled in a holistic manner. In this regard, it is crucial to address the direct and indirect relationships between PTG, PR, PC, and CR. Calhoun and Tedeschi proposed that the first step toward PTG is “cognitive engagement” (65). This reconstruction process that occurs after a traumatic event leads individuals to rethink repeatedly about the circumstances of the setbacks they have experienced, in the hope of giving it some meaning and build new life goals, which takes months or years (19). Since our survey was conducted in June 2021, more than 1 year after the initial outbreak, we can reasonably infer that college students have had a sufficient amount of time to adapt to such stressful environment and fulfill positive changes eventually. Indeed, the high PTG and PR scores in our sample (Median 2.62 and Median 2.44, respectively) confirmed the psychological rebound and recovery in the context of the pandemic.

This study focused on college students during the COVID-19 pandemic in the Chinese background. As shown in Table 1, the demographic analysis about individuals’ awareness of COVID-19 indicated that most of the sample hold a positive attitude toward the pandemic (as measured by Estimation score), such as paying more attention to their physical and mental health than usual (as measured by Concern score) and satisfying with the present prevention measures at present (as measured by Satisfaction score). These may provide ample evidence that when facing this ongoing trauma, Chinese college students still showcased hope and optimism. Although initially severely affected by the outbreak, China has since made significant progress in the prevention and control of the infection that causes COVID-19. To date, most Chinese students have been safely guarded benefit by the Home Quarantine Order and carried out Nucleic Acid Test screening, at the same time, colleges and universities have formulated flexible learning plans for students through online course training as appropriate to ensure normal teaching. In addition, Chinese colleges have conducted vigorous advocacy about the outbreak responses and reduced the panic caused by the fear on a variety of mass media platforms.

The current study found that all the hypotheses we initially proposed have been supported. To be specific, our findings prove that PTG has a direct and positive association with PR. PC and CR, respectively, mediated the relationship between PTG and PR. Thus, serial mediation existed among those variables: students with high-level PTG tended to report increased use of PC, which further facilitated their CR and subsequently, promoted their PR. As predicted, a positive correlation between PTG and PR was assessed, this result is basically consistent with Hypothesis 1 and with previous research (19), in which a positive relationship between PTG and PR was found. A previous study examined the relationship between PTG and PR of nursing university students after the COVID-19 alarm status in Turkey and found a predictive effect of PR on PTG (66). However, the present result indicates that PTG is a significant predictor of PR, which potentially provides a novel perspective of the impact factor studies of PR. PR, reflecting problem-solving ability or positive adaptation, is an individual’s ability to overcome adversities with positive developments. It has already been verified to enhance individuals’ understanding of happiness and promote mental health (3). PR is regarded as a mediator, moderator, or dependent variable in most previous studies. For instance, one study examined undergraduate and graduate students from China, Ireland, Malaysia, South Korea, United States and so on, and found that increased PR weakened the relationships between perceived stress and anxiety on sleep quality during the height of the COVID-19 pandemic (67).

Nevertheless, no study has further investigated the PR improvement mechanism among college students especially in the COVID-19 background. Specifically, PR can be promoted in different ways, such as providing a supportive social network (68), elevating general self-efficacy (23), enhancing active coping self-efficacy strategies (e.g., enhancing the perception that one is able to manage or recover from a stressful event), learning mindfulness skills (e.g., deep breath and focusing on the present moment), as well as nurturing a sense of purpose in life and the ability to find meaning (69–71). According to the dimensions of PTG, traumatic events may have caused individuals to undergo positive changes and improvement in interpersonal relationships, future possibilities, personal strength, mental state, and attitudes toward life. PTG differs from PR in that it is characterized by the gain of positive psychological benefits, whereas resilience is characterized by a return of the individual to its initial state (pre-crisis state) (19).

The present study proposed that positive gains of PTG may be correlated with the aforementioned factors from a different angle (23). Indeed, our findings are consistent with Hypothesis 2, 3, and 4 and previous studies, especially, confirming that serial mediation existed in the above relations in the context of the pandemic. In terms of the intermediary model, PC and CR, respectively, mediated the relationship between PTG and PR. Firstly, we found support for Hypothesis 2 that PC can partially and positively mediate the relationship between PTG and PR. This means that the effect of college students’ PTG on their PR is partly produced through PC. An individual with PC strategies can successfully evaluate and address difficulties in their lives. Based on a previous literature, a cross-sectional study was conducted using an online-based survey among university students during the official lockdown in Hungary when dormitories were closed, and teaching was conducted remotely. Results showed that among the domestic students, cognitive restructuring as a PC strategy was associated with lower levels of stress and anxiety (72). Also, PC is correlated to higher levels of positive cognitive and behavioral adjustments (73), which are representative of PTG in the face of stressful events. It has been reported that college students who were more optimistic and used positive coping methods were more willing to participate in social activities and manage the adverse impact of stress (74). Secondly, we found support for Hypothesis 3 that CR can partially and positively mediate the relationship between PTG and PR. This means that PTG strengthened PR via CR, a kind of ER strategies. Psychologists have theorized that ER bolsters PR by facilitating adaptive psychosocial processes (52, 75). More importantly, numerous studies have demonstrated that reappraisal is positively associated with PR (42, 76). CR is conceptualized as an adaptive strategy of positively reinterpreting a stressor to mitigate or control its emotional impact (77). One potential explanation for this finding is that more motivated individuals for positive growth prefer seek to implement efficient ways to adjust emotion and recontribute confidence when facing stressful situations (78, 79). Finally, for the first time, we found support for the serial mediation model of Hypothesis 4. College students who gained growth from traumatic events would be more easily to get recovery and adaption in the following crisis through emotional cognition reinterpreting, which is utilized based on a more positive coping strategy. Hence, it is essential to treat PC and CR which developed from PTG as viable options to promote PR among Chinese college students under trauma and adversity.

In the present study, the relationship between the PTG developed due to the COVID-19 pandemic and PR was dealt with in the context of PC and CR, which are discussed by considering the constructed models. However, the findings obtained from the present study need to be considered in the context of several limitations. On the one hand, all demographics and primary psychological variable assessment data were self-reported by students recruited by their teachers. Therefore, this data might have been affected by participants’ potential reporting bias. Future research should give sufficient consideration to the principle of voluntariness. On the other hand, due to the cross-sectional design, the current study did not allow us to establish definite inferences about a causal/directional relationship between the investigated variables. A prospective longitudinal design and experimental studies are required to demonstrate the causal linkage. During data interpretation, it is essential to consider that the mediator/moderator might be correlated with unobserved prognostic variables that may confuse the outcome. Therefore, future studies could employ a multimodal approach or assess the presence of other intermediary pathways with underlying effect to overcome this issue and enrich literature linking different variables.

That said, taken together, the present study contributes the literature on positive psychological resources among college students in China during the COVID-19 pandemic. Novel theoretical and empirical insights into the understanding of how PTG can facilitate PR have been provided. Findings show that students with post-traumatic growth developed more positive coping styles and cognitive reappraisal strategies, thereby encouraging them to maintain a higher level of psychological resilience. It is hoped that this study will stimulate further research and discussion concerning positive psychological growth. Notably, the mental health of college students is significantly affected due to the COVID-19 pandemic. They require the help and support of society, family, and colleges. What’s more, in order to improve a higher level of students’ mental well-being, government and schools ought to pay due attention to the specific aspects of resilience and collaborate to resolve this problem in order to provide high-quality psychological care to college students.

## Data Availability Statement

The raw data supporting the conclusions of this article will be made available by the authors, without undue reservation.

## Ethics Statement

The studies involving human participants were reviewed and approved by the Ethics Committee of Liaoning Normal University. The patients/participants provided their written informed consent to participate in this study.

## Author Contributions

Both authors listed have made a substantial, direct, and intellectual contribution to the work, and approved it for publication.

## Conflict of Interest

The authors declare that the research was conducted in the absence of any commercial or financial relationships that could be construed as a potential conflict of interest.

## Publisher’s Note

All claims expressed in this article are solely those of the authors and do not necessarily represent those of their affiliated organizations, or those of the publisher, the editors and the reviewers. Any product that may be evaluated in this article, or claim that may be made by its manufacturer, is not guaranteed or endorsed by the publisher.

## Footnotes

1
https://www.wjx.cn/
